# Scaling up access to oral rehydration solution for diarrhea: Learning from historical experience in low– and high–performing countries

**DOI:** 10.7189/jogh.03.010404

**Published:** 2013-06

**Authors:** Shelby E. Wilson, Saul S. Morris, Sarah Skye Gilbert, Emily Mosites, Rob Hackleman, Kristoffer L.M. Weum, Jillian Pintye, Lisa E. Manhart, Stephen E. Hawes

**Affiliations:** 1 Bill & Melinda Gates Foundation, Seattle, USA; 2 University of Washington Strategic Analysis Research and Training (START) Program, Seattle, USA

## Abstract

**Aim:**

This paper aims to identify factors that systematically predict why some countries that have tried to scale up oral rehydration solution (ORS) have succeeded, and others have not.

**Methods:**

We examined ORS coverage over time, across countries, and through case studies. We conducted expert interviews and literature and data searches to better understand the history of ORS scale–up efforts and why they failed or succeeded in nine countries. We used qualitative, pairwise (or three–country) comparisons of geographically or otherwise similar countries that had different outcomes in terms of ORS scale–up. An algorithm was developed which scored country performance across key supply, demand and financing activities to quantitatively assess the scale–up efforts in each country.

**Results:**

The vast majority of countries have neither particularly low nor encouragingly high ORS use rates. We observed three clearly identifiable contrasts between countries that achieved and sustained high ORS coverage and those that did not. Key partners across sectors have critical roles to play to effectively address supply– and demand–side barriers. Efforts must synchronize demand generation, private provider outreach and public sector work. Many donor funds are either suspended or redirected in the event of political instability, exacerbating the health challenges faced by countries in these contexts. We found little information on the cost of scale–up efforts.

**Conclusions:**

We identified a number of characteristics of successful ORS scale–up programs, including involvement of a broad range of key players, addressing supply and demand generation together, and working with both public and private sectors. Dedicated efforts are needed to launch and sustain success, including monitoring and evaluation plans to track program costs and impacts. These case studies were designed to inform programmatic decision–making; thus, rigorous academic methods to qualitatively and quantitatively evaluate country ORS scale–up programs might yield additional, critical insights and confirm our conclusions.

Oral rehydration therapy (ORT) for dehydrating diarrhea came into routine use at Bangladesh’s Cholera Research Laboratory (now ICDDR,B) in 1969. Nine years later, the World Health Organization recommended a standardized version of the therapy – Oral Rehydration Salts, or ORS – for all acute watery diarrhea in children [1]. However, on average between 2006 and 2011, only one third of children with diarrhea in developing countries received ORS [2].

These low rates of ORS use are surprising given the emphasis given to this product in the years after its introduction. The product was one of the foci of UNICEF’s “GOBI–FFF” selective primary health care strategy of 1982 (the “O” in GOBI refers to ORS) [3]. In the mid–1990s, ORS was similarly made the keystone of diarrheal disease management in WHO’s “Integrated Management of Childhood Illness” initiative [4], and *The Lancet* child survival series of 2003 identified it as the single intervention available at that time with the greatest potential to save lives [5]. Yet throughout this period, ORS gained ground at a rate of just 0.6 percentage points per year (analysis based on data available online from UNICEF [6], MEASURE DHS [7] and other national surveys).

Twenty countries (out of a total of 96 with data from the standard surveys series that track ORS coverage, including Demographic and Health Surveys (DHS) [6] and Multiple Indicator Cluster Surveys (MICS) [8]) have entirely failed in promoting rational diarrhea management, with less than 25% of pediatric diarrhea episodes treated with ORS. Yet many other countries have done much better, with 29 countries using ORS in one half or more of all episodes, and eight countries using ORS in two–thirds or more of all episodes. As can be seen in the map (**Figure 1**), by this criterion, high performing countries are found in every region of the world. The vast majority of countries in sub–Saharan Africa, as well as many Indian states, have neither particularly low nor encouragingly high rates of ORS use, but rather fall somewhere in the middle.

**Figure 1:**
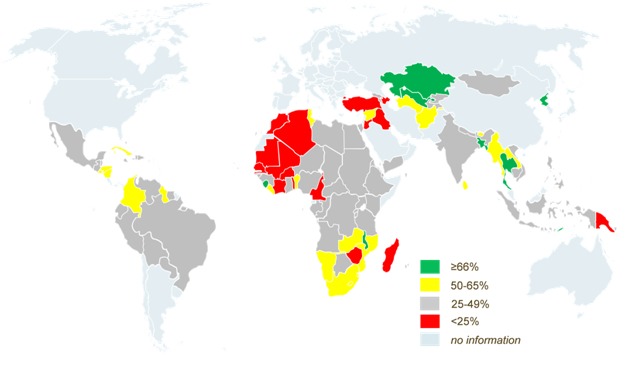
Map of ORS use rates around the world. (Data sources: Demographic and Health Surveys, Multiple Indicator Cluster Surveys and national survey series).

The objective of the present study was to address the question: Why have some countries succeeded in scaling up ORS for diarrhea, when others tried and failed? We hypothesized that countries would be more likely to have been successful in scaling up ORS if they simultaneously: 1) made significant improvements to the standard product offering, including lowering the price; 2) conducted wide–reaching marketing campaigns; 3) acted to remove regulatory barriers to the sale and promotion of ORS in the private sector; 4) improved private provider knowledge of rational diarrhea management; 5) improved public provider knowledge of rational diarrhea management; 6) made a concerted effort to improve the regularity of supply; and 7) mobilized partner funding as well as taking country ownership of the program. Some of these concepts have been examined in previous case studies [1,9] but we are not aware of any other work that has examined all of them simultaneously. To test these hypotheses we reviewed peer–reviewed articles and grey literature and conducted key informant interviews to learn about the history of ORS scale–up in each of the selected countries, completed qualitative case studies using information obtained from literature searches and interviews, and quantitatively analyzed the findings from these sources.

## METHODS

We categorized every country with ORS coverage data into three categories: countries that exceeded 50% coverage for ≥5 years and now have achieved >66% coverage were defined as “sustained success” (**Table 1**); countries that achieved >50% coverage at one point in time, then saw reductions in coverage of at least ten percentage points were defined as “unsustained success” (**Table 2**); and countries never reaching ORS coverage rates of 25% despite targeted scale–up efforts were defined as “non–starter (failure)” (**Table 3**).

**Table 1:** Countries meeting the criteria for “sustained” success

Country	First year ORS coverage >50%	Most recent year ORS coverage >50%	Most recent coverage measured level	Total number of years coverage >50%
Bangladesh	1994	2011	77.6	17
Democratic People's Republic of Korea	2000	2009	74.0	9
Malawi	2001	2010	69.1	9
Sierra Leone	2003	2010	72.6	7
Thailand	1996	2006	68.3	10

ORS – oral rehydration solution

**Table 2:** Countries meeting the criteria for “unsustained success”

Country	Maximum ORS coverage reached	Year maximum coverage reached	Subsequent decline in coverage	Most recent year ORS coverage reported*
Kazakhstan	73.3	2006	11.5	2011
Lesotho	54.5	2000	12.1	2004
Mongolia	55.9	2000	17.9	2005
Swaziland	85.7	2007	28.1	2010
Trinidad and Tobago	52.3	1987	20.3	2000
United Republic of Tanzania	57.6	1992	13.9	2010

ORS – oral rehydration solution

*At time of classification for this study.

**Table 3:** Countries meeting the criteria for “non–starters”

Country	Maximum ORS coverage reached	Number of data points
Burkina Faso	21.2	5
Cameroon	21.9	5
Chad	23.4	4
Côte d'Ivoire	22.7	5
Madagascar	23.1	4
Mali	15.7	3
Mauritania	23.3	2
Morocco	24.4	3
Senegal	22.4	5
Togo	20.2	5
Turkey	15.9	2
Zimbabwe	20.9	3

ORS – oral rehydration solution

We did not expect coverage of ORS in any country to exceed 75% because the average duration of an episode of pediatric diarrhea is 3.1 days, and approximately 25% of all episodes are of such brief duration (or mild presentation) that they do not significantly contribute to mortality and morbidity [10]. Caregivers are therefore not motivated to seek treatment outside the home. Although ORS is recommended for all episodes [2], we view a use rate of 75% as the maximum level that can be achieved at a population level.

Nine countries were purposively selected for in–depth case studies, including Bangladesh [11], Guyana [12], India [13], Madagascar [14], Malawi [15], Senegal [16], Sierra Leone [17], Tanzania [18], and Trinidad and Tobago [19]. These included all of the possible sustained successes except for the Democratic Republic of Korea, where we knew that we would not be able find relevant information, and Thailand, which was excluded arbitrarily. We matched these successes to one “non–starter” country which was geographically close by and plausibly comparable in terms of size and health system organization. India was included as a historic comparator for Bangladesh because it was a “non–starter” until 2005, even though coverage has improved since that time; because of its subsequent change in status, when we scored this country for the quantitative analysis we only considered events up to 2005. Tanzania was studied as an interesting case of an “unsustained success” which could reasonably be compared to both Malawi (a “sustained success” scale–up country) and to Madagascar (a “non–starter”). In order to include a pair of case studies from the western hemisphere – where no country has reached our standard of “sustained success” – we included Guyana, where coverage reached 49.8% in 2009, and compared it to Trinidad and Tobago, a clear “unsustained success”. We conducted similar case studies of zinc uptake in four countries, including Bangladesh [20], Madagascar [21], Nepal [22], and Tanzania [23]. However, because there is more information and longitudinal data, we focused this manuscript on ORS scale–up.

In each country, literature reviews and expert interviews were conducted to better understand the history of ORS scale–up efforts and why they failed or succeeded. The review included peer–reviewed articles, conference presentations, and grant reports available to the Bill & Melinda Gates Foundation and partners. An initial search was conducted in PubMed in April 2012 and the titles and abstracts of all the retrieved citations were examined for relevance to ORS scale–up efforts in each country. Following the initial search, multiple searches were conducted in PubMed and Google through October 2012 to identify studies and reports related to ORS scale–up in the selected countries. The search strategy was restricted to documents written in English and French. The following keyword terms (in English) were used with each country: ORS, ORT, and diarrhea.

Additional articles and reviews related to the relevant topics were identified by hand–searching the references in the articles and reports identified through the search engines. We also sought additional literature from the United Nations Children’s Fund [24], the World Health Organization [25], and the World Bank [26] websites. Reports from USAID (United States Agency for International Development)–funded projects were retrieved from the USAID Development Experience Clearinghouse [27].

Data for a wide range of monitoring and impact evaluation indicators in the areas of population, health, and nutrition were obtained from Demographic and Health Surveys [28], Multiple Indicator Cluster Surveys [29], and Malaria Indicator Surveys [30]. Information about the characteristics of health facilities and services available in a country was obtained from Service Provision Assessment Surveys [31]. Data on drug prices, availability and affordability were obtained from the World Health Organization/Health Action International surveys and reports [32].

The “snowball” technique was used to identify informants. An initial list of potential key informants was generated through personal communication with experts in the field of diarrhea management, with the aim of interviewing individuals from a range of sectors to provide a broad range of perspectives. Potential key informants from governments, donors, multilateral and bilateral organizations, non–governmental organizations (NGO), the local private sector, and academic and clinical institutions were contacted individually via email to request telephone or, in some cases, in–person interviews. Between April and September 2012, key informant interviews were conducted with 58 experts (**Table 4**) to understand what efforts were made improve ORS use in the past, and how, with the benefit of hindsight, well–informed observers think these efforts could have been better designed in each of the nine countries. The interviewers took notes during and after the interviews to document the key informants’ responses.

**Table 4:** Dimensions of implementation and their scales

Dimension	Definition	Scale
Political stability	The degree to which a country has had minimal political conflict, civil unrest and/or violence	High–Medium–Low
Natural disasters	The number of natural disasters experienced by the country during the ORS or zinc scale–up time period	High–Medium–Low
U5 mortality	U5 No. deaths	U5 No. deaths
U5 deaths due to diarrhea	U5 No. deaths due to diarrhea	U5 No. deaths due to diarrhea
Vaccine coverage	The immunization rate	% DTP3
Zinc introduction	Whether the country has introduced zinc	Y/N
Private sector share	Of those seeking care for diarrhea, % going to private sector	% going to private sector
USAID recipient	Whether the country has received funding from USAID	Y/N
Home–based solutions promotion	Whether the country initially promoted home–based sugar–salt solutions	Y/N
IMCI country	Whether the country has introduced IMCI	Y/N
Decentralization of responsibility not funding	Whether the country has decentralized responsibility without also decentralizing funding	Y/N
Degree of collaboration across government, private, public	Extent to which partners worked together on diarrhea case management	HIGH – Multiple types of partners involved and collaborating MEDIUM – Multiple types of partners involved but tense relations OR few partners but strong relations LOW – No partnerships, or a few non–collaborative ones
Female literacy rate	Self–explanatory	% females literate
Diarrhea care–seeking	Degree to which caregivers sought treatment for diarrhea when their child fell ill	% seeking care
Reach of health system	Quality of the health infrastructure	HIGH – Infrastructure has broad reach and HCW capacity appropriate for pop size MEDIUM – Broad reach OR appropriate HCW capacity LOW – Insufficient reach and poor capacity
Surface area	Self–explanatory	square km
Population	Self–explanatory	No. people
		
SCALE–UP INDICATORS		
Improved product, including pricing	Degree to which scale–up attempt improved the ORS or zinc product, including making it affordable	HIGH – Price not a barrier to purchase; consumer research conducted to determine preferences MEDIUM – Generally strong product, but price or consumer research sub–optimally conducted LOW – Pricing and product not informed by any prior information
Marketing campaign	Degree to which scale–up attempt conducted a successful direct–to–consumer marketing campaign	HIGH – Multi–channel, researched campaign of sufficient duration; consumer demand increased MEDIUM – Multi–channel or of long duration; high knowledge low utilization LOW – Little impact on awareness and/or use after the campaign
Regulatory approval	Whether regulatory hurdles were overcome	Y/N
Improved private provider knowledge	Degree to which campaign successfully got private providers to recommend ORS and/or zinc	HIGH – For areas where high use of private providers, specific interventions targeting private providers; for others, inclusion in outreach; positive impact on provider recommendations MEDIUM – Inclusion in outreach, awareness but not impact LOW – Not included in outreach
Improved public provider knowledge; increasing supportive supervision	Degree to which campaign successfully got public providers to recommend ORS and/or zinc	HIGH – For areas where high use of public providers, specific interventions targeting private providers; for others, inclusion in outreach; positive impact on provider recommendations MEDIUM – Inclusion in outreach, awareness but not impact LOW – Not included in outreach
Increasing availability of supply	Degree to which scale–up including local manufacturing, and consistent availability of quality product	HIGH – Few stockouts, local supplier, private and public MEDIUM – Modest number of stockouts, foreign supply, private or public LOW – Low availability
Financing of scale–up	Degree to which countries successfully began to own scale–up and both scale–up and maintenance had sufficient funds	HIGH – Partners contribute but country assume ownership; sufficient funding in volume and duration MEDIUM – Partners contribute or country ownership, but funding insufficient LOW – Few contributors; insufficient funding

An interview tool (Online Supplementary Document(Online Supplementary Document)) was developed to guide the discussion and to ensure that the primary questions were answered. Semi–structured interviews were used to elicit open–ended responses. Not all interviewees were asked exactly all the same questions despite utilization of the same interview guide, given the semi–structured nature of the interviews, and that differing perspectives were sought from each type of partner. We circulated the draft case studies to local key informants with requests for their review and comment. The case studies were finalized following this validation.

The team used qualitative, pairwise (or three–country) comparisons of geographically or otherwise similar countries that had different outcomes in terms of ORS scale–up. In addition to the qualitative pairwise (or three–country) comparisons of country case studies, the scale–up efforts in each country were quantitatively assessed, based on a numeric scoring of country performance across various key supply, demand and financing activities. For each case study, we scored the country on their efforts along seven dimensions of implementation, with a range of scores from 0 (low/no effort) to 2 (high effort). The sum of these scores yields an implementation score ranging from 0 to 14. The seven dimensions of implementation included the following: made significant improvements to the standard product offering, including lowering price; conducted wide–reaching marketing campaigns; acted to remove regulatory barriers to sale and promotion of ORS in the private sector; improved private provider knowledge of rational diarrhea management; improved public provider knowledge of rational diarrhea management; made a concerted effort to improve the regularity of supply; mobilized partner funding as well as taking country ownership of the program.

The quantitative portion of evaluation was conducted through a scoring algorithm. Scoring was made as consistent as possible across countries by developing precise definitions of what was included in “0”, “1” or “2” scores for each dimension (**Table 5**). Since not all countries had the same data availability, scoring definitions generally had 2–3 components so that countries with non–equivalent information could still be classified. After scoring each component, aggregate scores were calculated for each country by summing across components. A statistical test was conducted with the aim of rejecting the hypothesis that there was a trend in the aggregate scores across the three categories of countries (H1: score_sustained_success_>score_unsustained_success_>score_non–starter_). The test used was Cuzick’s non–parametric trend, which is an extension of the Wilcoxon rank–sum test [33]. Given that the scoring criteria demanded a certain degree of subjectivity in order to classify countries with different types of data, we urge caution in interpreting these results.

**Table 5:** Number and type of key informants accessed for each country

Country	Government		Donor		Multilateral and bilateral		NGO	Local private sector		Academia			Clinical	Total
Bangladesh		0		1		0	2		2		1	0		6
Guyana		1		0		1	0		0		0	1		3
India		0		0		0	3		1		0	0		4
Madagascar		1		0		0	7		0		0	0		8
Malawi		1		0		1	4		0		0	0		6
Senegal		0		1		1	1		0		0	1		4
Sierra Leone		1		1		0	5		0		0	0		7
Tanzania		1		0		2	7		1		0	0		11
Trinidad and Tobago		0		0		3	1		0		0	2		6
Multi–country input		0		0		0	3		0		0	0		3
Total		5		3		8	33		4		1	4		58

NGO – Non-governmental organization

## RESULTS

### Country comparisons

**Figure 2:**
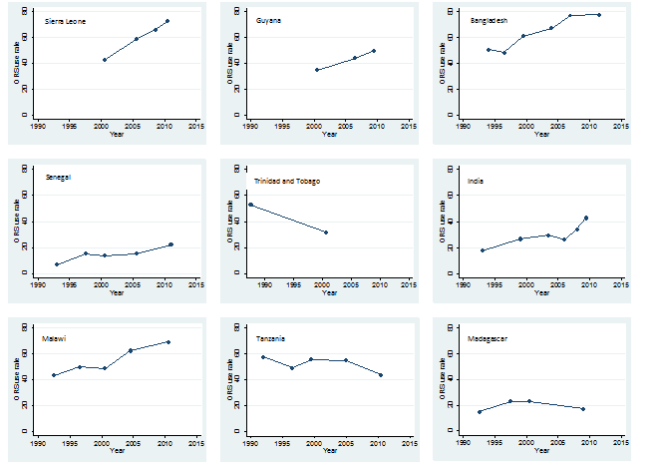
Oral rehydration solution (ORS) use rates by country, 1987-2011.

Country comparisons are presented in **Figure 2**.

**Comparison 1: Sierra Leone vs Senegal.** The West African country of Sierra Leone, mired in civil war from 1991–2002, is a perhaps unexpected example of sustained, successful scale–up. Even before the civil war, Blue Flag Volunteers (BFVs) were trained to promote hygiene and treat diarrhea with ORT. During the conflict, which displaced as many as 2 million out of the 5.5 million population, ORS was pushed heavily on displaced populations in camps. After the civil war, the primary health care system was “reinvented”, with multi–donor support. Recurrent cholera outbreaks were managed with ORS and efforts were made to ensure that the supply chain was maintained at each level: the Ministry of Health and Sanitation increased the intensity of tracking ORS distribution, districts and communities were in charge of ordering supplies, and peer supervisors facilitated re–stocking ORS in Community Based Distributors’ (CBDs) kits. The population learned about the product via interpersonal communications with very active community promoters (BFVs and or CBDs). Integrated community case management of childhood illness was introduced to some districts in late 2000s and free health care for pregnant and lactating women and children under–five was introduced in 2010 [34–37].

Senegal is a West African country of 13 million people where ORS scale–up efforts have not succeeded (“non–starter”). The country is very stable, with a well–organized health system and relatively good infrastructure. The United States provided over half of all bilateral aid for health (basic) in the 2000s. USAID has been promoting ORS in Senegal since 1985, through multiple, successive, standalone “Technical Assistance” programs in “USAID regions” in the West – including PRITECH (1985–1993), BASICS (1994–2006), Child Survival Program (1998–2002), Fatick Partnership (2007–2009), Community Health Program (2006–2011). UNICEF supplied all ORS in the country until 2000 and Integrated Management of Childhood Illness (IMCI) was introduced in the 2000s, but the guidelines resulted in no change in clinical practice. The “health hut” program was launched in the late 1990s, including activities to promote both ORS and home fluids (including sugar–salt solution). However, ORS was not widely available in Senegal due to a weak distribution system that caused frequent shortages and stock–outs: availability of essential drugs was highest at the storage facilities and decreased at the more peripheral health facilities [38]. There are now 1600 health huts in Senegal, but they recover the full cost of medicines and, when surveyed, few (6–47%) health providers knew how to correctly look for signs of dehydration [39].

There are four key lessons from this comparison. First, interpersonal communications led to universal familiarity with ORS in Sierra Leone, whereas 41% of the population in Senegal still does not know about this product [40]. In Sierra Leone, emergencies provided an opportunity for boosting confidence in the product, and its purpose (rehydration, not symptom relief) is understood and appreciated. This understanding has been undermined in Senegal by mixed messaging about the benefits of ORS vs sugar–salt solution.

Second, basic supply chain management kept ORS widely available in Sierra Leone; in Senegal, it was unavailable in the private sector because ORS does not have a “visa” required to register drug products [36] and therefore could not be sold as a medicine, and public sector availability was also inconsistent. Further, pricing favored ORS in Sierra Leone, where the product is cheaper than in Senegal and where the cost of sugar made home–produced alternatives unaffordable (also not the case in Senegal). Finally, the regional stand–alone project approach of USAID in Senegal appears to have been too limited in scope and scale to increase ORS usage, compared to the “national reconstruction project” in Sierra Leone, launched with lots of donor support to restore infrastructure and health services, and through which a comprehensive community case management program was created. Key informants suggested that there was limited government spending on scale–up efforts in Senegal during the period under consideration.

**Comparison 2: Guyana vs Trinidad and Tobago.** Guyana is a “sustained success” country of just 742 thousand people, in South America but culturally of the Caribbean. The country is susceptible to flooding and experienced a cholera epidemic in 1992 [41]. After the 1992 cholera epidemic, Guyana created a national policy for the treatment of diarrhea, including ORS. The Ministry of Health purchased large quantities of ORS, including pre–mixed liquid, and stockpiled product for disasters. The country’s dense network of public health centers and health posts was supplemented by 287 Community Health Workers who extended the reach into deep rural areas and demonstrated correct use of ORS. According to key informants, the Ministry of Health conducted yearly, seasonal marketing for ORS, using newsprint, television, and radio and made ORS available free in the public sector.

Trinidad and Tobago is an “unsustained success” Caribbean country of 1.2 million people and is relatively wealthy with abundant oil and gas. Key informants recalled that a single local champion established Oral Rehydration Units in three major hospitals/health centers in the capital in 1981, in conjunction with an early 1980s ORS marketing campaign funded by the International Development Research Center (IDRC, US$ 132 thousand), which used a logo on printed materials and also ran radio and television spots. One radio station hosted a call–in program with health care providers and focused on diarrhea prevention. Key informants suggested that the campaign was intended to be both relevant to and empowering for mothers, and relied on mother–to–mother interpersonal communication to disseminate messages. Messaging switched to promoting “rehydration”, and there was even distracting debate over merits of coconut water as a source of liquidand electrolytes [42]. Ultimately, scale–up of water and sanitation improvements has been associated with a major reduction in diarrhea incidence; diarrhea is no longer seen as national priority since diarrheal disease accounted for less than 1% of deaths among children under–five in 2010 [43].

This comparison illustrates that ORS is very hard to promote if it is not epidemiologically relevant. Although both countries experienced declines in mortality to low levels, ORS retained relevance in Guyana because of repeated outbreaks of diarrheal disease after flooding. This comparison underscores the essential nature of broad national buy–in. The program in Trinidad and Tobago relied on one person and external funding, whereas Guyana has embraced ORS as part of its commitment to universal primary health care, with the government even taking on responsibility for regular communication campaigns. Finally, clarity of message is essential. In Trinidad and Tobago, the messaging was inconsistent between ORS, breastfeeding, and even coconut water, whereas Guyana made a strong and lasting commitment to ORS, and folded this seamlessly into integrated programs such as Integrated Management of Childhood Illness (IMCI).

**Comparison 3: Malawi, Tanzania and Madagascar.** Malawi is a land–locked country in Eastern/Southern Africa with 16.3 million people and successfully sustained scale–up. The country has never had significant civil unrest or natural disasters. Malawi implemented a National Control of Diarrheal Disease Program in 1985 and stopped promoting sugar–salt solution in 1989, using multiple channels to popularize ORS (so that 90% of the population was familiar with it by 1992). Integrated Management of Childhood Illness (IMCI) was rolled out comprehensively to all districts and a cadre of “Cholera Assistants” was instituted in the 1970s. These were developed into Health Surveillance Assistants now present in all “hard–to–reach” communities of the country [44]. From the early 2000s, USAID funded Population Services International (PSI) to socially market branded ORS, achieving universal recognition, and massive penetration of pharmacies and retail outlets. The product was free in the public sector and heavily subsidized in the private sector [45].

Tanzania has 46.9 million people and was classified as an “unsustained success” for ORS scale up because it achieved greater than 50% coverage and then declined. Tanzania implemented a National Control of Diarrheal Diseases Program in the 1980s, with “Diarrhea Treatment Corners” in health centers and hospitals and achieved 93% familiarity with ORS by 1991 [46]. The IMCI Strategy was gradually rolled out and evidence from a local trial showed that IMCI did not improve ORS use [47]. Although ORS was widely available in the public sector in 2006 [48], availability appears to have fallen (to 57.4%) by 2009 for unclear reasons [49]. A local producer (Shelys) did little to stimulate demand, relying on public tenders; the second local supplier went bankrupt in 2008. USAID–funded outreach to pharmacists and Accredited Drug Dispensing Outlets (ADDOs) resulted in good availability from the early 2000s, but conducted little marketing beyond these segments [50]. There was little direct–to–consumer marketing of ORS in Tanzania except through USAID’s Point–of–Use Water Disinfection and Zinc Treatment (POUZN) project (2005– 2010) which was all non–branded promotion and time–limited [51]. Unlike Malawi, Tanzania lacks a community health worker program at scale. ORS is not always free in the public sector and there is no subsidy.

Madagascar is an island of 22.0 million people and was classified as a “non–starter” for ORS scale–up because it never reached greater than 23.1% coverage. It has experienced a relentless series of natural disasters, and, in 2009, widely perceived to have been unconstitutional transfer of power. There was no ORS in country at all until 1988. UNICEF then supported a local producer with limited capacity, which eventually went out of business, leaving the country wholly dependent on imported supply. While many countries import health products, key informants suggested that the frequency with which Madagascar experienced natural disasters and the political turmoil that led to import disruptions resulted in poor accessibility of ORS. Early radio campaigns promoted both ORS and home–made sugar–salt solution. USAID supported co–packaged products only through the POUZN Project from 2008 to 2010 but diverted support to non–government entities following a transfer of power, which severely weakened program efforts to scale–up ORS [52].This diversion likely contributed to the poor penetration of programs for training public providers and community–based distribution, which reached <10% and 15%, respectively, of the country.

This comparison reveals that once universal familiarity with ORS is achieved, availability is extremely important. Malawi achieved very high levels of availability in both public and private sectors, whereas in Madagascar, uptake has been crippled by lack of availability. In Tanzania, the weakening public sector supply chain may explain recent declines in ORS use. An organized cadre of trained community–based distributors can greatly extend the reach of the public sector to achieve market penetration at scale. It is possible to capitalize on floods and cholera epidemics to increase familiarity with and trust in ORS, but countries enmeshed in political unrest are not conducive to ORS scale–up. Reliance on a single donor is risky because support may be abruptly terminated before programs are mature.

**Comparison 4: Bangladesh vs India.** Bangladesh, a South Asian country of 161 million people, was the first country in the world to accumulate large–scale experience using ORT. It is home to the world renowned International Centre for Diarrhoeal Disease Research (ICCDR,B). ICDDR,B developed ORT and continues to research and promote the approach, modeling successful control of diarrhea mortality. In 1981, the government created the National Oral Rehydration Project and distributed packets of ORS to health centers in 100/509 sub–districts [53]. Between 1980 and 1990, BRAC (formerly Bangladesh Rural Advancement Committee) trained 12 million women (approximately half of all women in the country) in preparation and use of sugar–salt solution, and still trains community health workers [51]. Bangladesh explicitly switched from promotion of sugar–salt solution to promotion of ORS. Starting in 1985 Population Services International (PSI) and, later, the Social Marketing Company (SMC) promoted branded ORS through multi–channel social marketing, spending US$ 1 million/year and capturing 80% of the market. In the early 2000s, SMC built its own manufacturing facility and services 220 000 retail outlets [54]. According to key informants, in addition to SMC, there are now 30–40 ORS suppliers. ORS is supplied for free in the public sector and is very cheap in the private sector (US$ 0.06) [52]. Bangladesh relied on the family unit to sustain ORS use – the majority of mothers now educate their children on ORS, removing the need for repeated marketing campaigns. The most recent DHS survey provides clear evidence of successful ORS scale–up efforts: 77.6% of recent diarrhea episodes were treated with ORS (and 40.8% were treated with zinc), and only 2.0% of all under–five deaths were attributed to diarrhea [55].

India’s 1.2 billion people reside in 28 states and seven Union Territories, each with hugely different public health systems and health outcomes. India was classified as a “nonstarter” through 2005 then had dramatic increases in coverage. India’s multiple large scale government programs – including Diarrheal Disease Control Program (1978), Child Survival and Safe Motherhood Program (1992), Reproductive and Child Health Program (1997), and National Rural Health Mission (2005) – have resulted in high ORS availability in the public sector and training and stocking of outreach workers such as Anganwadi Workers and, more recently, Accredited Social Health Activists (ASHAs). In the 1980s, UNICEF promoted sugar–salt solution [56], which is still more familiar and more widely used than ORS. There is no national consensus in favor of ORS. The Program for Advancement of Commercial Technology–Child and Reproductive Health (PACT–CRH), a US$ 30 million USAID project, began strong promotion of ORS in the mid–2000s, with celebrity partnerships, media, home visits, and free samples [57]. India’s vibrant pharmaceutical industry actively markets antibiotics to private providers, who command at least two–thirds of the market [58]. Public health detailing to private providers has never been done at scale.

This comparison underscores that even in countries where the market for diarrhea treatment is dominated by the informal private sector; it is possible to achieve high levels of use of ORS by changing social norms. It is likely that intensive interpersonal communication is a critical part of this behavior change, and it is also important to directly reach frontline providers at scale. Conversely, an excessive emphasis on public sector providers, in a context where, according to key informants, the public sector is under–valued and under–utilized, is not likely to be very effective, at least in the short term. Branded marketing can be very helpful, but must be explicitly directed at the mass market (India’s most successful Electral brand has never been marketed as a product intended for self–treatment).Unambiguous messaging that home–made sugar salt solution is not an adequate substitute for ORS is critical for successful scale–up. Bangladesh benefited from the leadership of a highly respected local institutional champion for ORS (ICDDR,B) and managed to create a broad alliance of major local stakeholders. In India, the major champions of ORS have been mostly external, and there is no equivalent of BRAC that reaches beyond the public sector.

### Quantification of scale–up factors analysis

**Figure 3:**
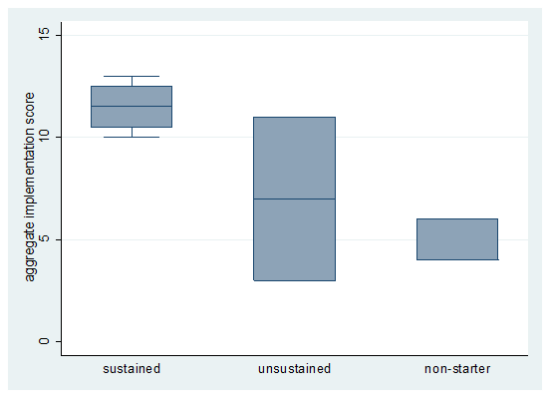
Aggregate implementation scores, by country scale-up type. Box plot with whiskers from minimum to maximum scores for the “sustained success” countries. The whiskers indicate the range of the data and are represented as vertical lines ending in a small horizontal line. The median and the interquartile range (IQR) were used to construct each box. The horizontal bar in the middle is the median score and the height of the box is equal to the IQR, drawn so that it starts at the 25^th^ percentile (lower quartile value) and stops at the 75^th^ percentile (upper quartile value).

There was strong evidence that the aggregate scale up scores were more favorable in the countries that achieved more sustainable scale–up, ie, countries that exceeded 50% coverage for ≥5 years and now have achieved >66% coverage (*P* = 0.042, Cuzick’s non–parametric test for trend; **Figure 3**). Although the very small sample size suggests caution when interpreting between group differences in individual implementation categories (**Table 6**), there is overwhelming evidence that the four successful countries all implemented well–researched, multi–channel communications campaigns, whereas this was not done at all in the two “non–starter” countries, and only to a very limited extent in the three “unsustained success” countries. The next strongest association was with the financing of scale–up, where the more successful countries were more likely to have mobilized substantial funds from partners and taken significant ownership themselves.

**Table 6:** Scores given to each country for each scale–up criterion

Scale–up indicators	Bangladesh	Malawi	Sierra Leone	Guyana	Tanzania	Trinidad & Tobago	Senegal	India*	Madagascar
Improved product, including pricing	2	2	1	1	0	1	1	1	1
Marketing campaign	2	2	2	2	0	1	0	1	0
Regulatory not a barrier	2	2	2	2	2	2	2	2	2
Improved private provider knowledge	1	2	0	1	1	2	0	0	1
Improved public provider knowledge; increasing supportive supervision	2	1	2	2	0	2	1	1	1
Increasing availability of supply	2	2	2	1	0	1	0	1	1
Financing of scale–up	2	1	1	2	0	2	0	0	0
TOTAL	13	12	10	11	3	11	4	6	6

*Up to 2005

## DISCUSSION

Since UNICEF placed ORS at the heart of its “GOBI–FFF” strategy in 1982, usage of ORS has increased slowly but steadily, suggesting a systematic response to public health recommendations [1]. Despite global efforts over the past four decades, however, current ORS use rates in the developing world are surprisingly low. While some countries have not succeeded in promoting rational diarrhea management and others have done much better, most countries are somewhere in the middle. This exercise was designed to explore the root causes of this diversity in ORS use, particularly focusing on direct and indirect contributors to ORS scale–up in countries with very high and very low ORS use rates.

Our initial hypotheses were mainly confirmed – that is, countries were more likely to have been successful in scaling up ORS if they (i) ensured both broad national buy–in and collaboration between government, non–government and private sectors; (ii) made significant efforts to synchronize demand generation, private provider outreach, and public sector work; and (iii) the context at the time of the interventions was conducive to scale up, including funding directed to ORS scale–up and the absence of political turmoil. We also drew some preliminary conclusions about specific elements of scale–up programs, though we believe these will need further validation. While these concepts are not entirely original and novel, we are not aware of any other work that has examined all of them simultaneously.

Key informant interviews suggested that partner collaboration was especially critical to success in Sierra Leone, Bangladesh and Guyana. The governments in all three countries collaborated with a range of development partners in the public and private sectors, reducing potential for “donor dependency”. Senegal’s reportedly weak government engagement in the early stages of ORS scale–up efforts and reliance on USAID were both cited by key informants as drivers of continuing low ORS use rates. Similar findings were reported from a case study in Philippines as our findings from Trinidad and Tobago: hospital admissions attributable to severe diarrhea declined and ORS became less epidemiologically relevant as income and sanitation improved [1].

The results of the quantification of scale–up factors analysis, despite relying heavily on qualitative information, suggested a correlation between high ORS use rates and synchronicity of demand– and supply–side interventions. All four “sustained successes” (Sierra Leone, Guyana, Malawi, and Bangladesh) focused on community–level delivery (although operationalized in very different ways), promoted ORS through health worker communications and mass media, provided ORS free of charge in public sector and had secure supply of the product. Malawi and Bangladesh have historically had robust community– and household–level interventions that highlight the importance of interpersonal communications to increasing uptake of ORS, and ensuring availability in areas close to where people live. Malawi also utilized tracking mechanisms to reduce stock–outs, and Bangladesh had a sales force for private sector outreach. “Non–starter” countries generally lacked coordinated, sustained efforts to improve supply and demand, and efforts were reportedly hindered by poor country ownership and insufficient financing. For example, the ADDOs in Tanzania can extend the reach of drug shops to rural areas, but given the vast distances in the country, gaps remain for certain segments of the population. Both Madagascar and, more recently, Tanzania struggled to maintain product availability at public health facilities, which may have contributed to low ORS use in those countries.

One of the most surprising findings was the importance of the context in which the scale–up efforts were implemented. The case studies show how clearly it played a role in the outcomes of scale–up efforts, and how there is no “one–size–fits–all” approach or program for ORS. The case study of Madagascar demonstrates how difficult it is to implement successful treatment programs when the health system is passing through a period of acute destabilization. This highlights the importance of fully understanding the risks of working in potential high–impact, volatile countries, where the need is great but the risk of program disruption is very high. However, we should note that many of the countries that we included in the case studies were relatively stable compared to Madagascar, so further studies to support or negate this hypothesis, and to better understand how to successfully operate in high–risk, high–need areas, will be critical moving forward.

We identified four other findings that merit further investigation. First, ambiguous messaging about the relative value of ORS vs home–made sugar–salt solution can stifle ORS utilization. The definition of “oral rehydration therapy” changed four times within a decade [1], and may have had a lasting impact on clarity of communications about the gold standard for treatment of diarrhea. In Tanzania and India, both of which have a vibrant pharmaceutical market, the lack of focus on ORS messaging has allowed this product to be displaced by anti–diarrheals and antibiotics. In contrast, unambiguous messaging, that home–made sugar salt solution is not an adequate substitute for ORS, is critical for successful scale–up. Bangladesh switched from promotion of sugar–salt solution to promotion of ORS and the recent estimates suggest that ORS packets were used in nearly 80% of under–five diarrhea episodes in 2011. Second, rigorous commercial marketing approaches should be combined with effective interpersonal communication. An earlier case study of ORT success in Egypt highlighted the importance of utilizing scientific evidence with consumer and market research in crafting relevant and appropriate messages [8]. Commercial partners who truly understand marketing, and favor branded over generic marketing, can team up with groups providing interpersonal communication delivered in the home or through self–help groups. Third, it is clear that the market for diarrhea treatment products can be highly price sensitive (eg, pricing was a barrier in rural Madagascar). Thus partners must work to bring down prices in countries where they remain stubbornly high. Finally, support to the private sector needs to articulate a clear path towards the creation of a sustainable market. Subsidies for ORS may undermine sustainable markets, thus business models that encourage suppliers’ reliance on external support risk being counter–productive in the long term. Few donor agencies recognize the importance of these domains of health services delivery to readily invest in them; public–private partnerships could lend support for this concept.

We found little information on the cost of scale–up efforts, primarily because this information has not historically been well–documented. We know that scale–up was done sustainably (and presumably affordably) in the five “successful” (>66% coverage) countries studied, and we have some anecdotal evidence from interviews and reports from major funders, but this is a gap in knowledge within the global community. To address this gap in future work, donors and governments would be wise to develop a rigorous monitoring and evaluation plan to track the impact of their investments and begin to improve our understanding of cost and sustainability.

There are several important limitations of this study. These case studies were initially conceived to inform programmatic decisions for the Bill & Melinda Gates Foundation; we recognize that the methods used did not emphasize repeatability as strongly as they would have done had our initial purpose been to publish these results in an academic journal. However, as important results came to light through this work, sharing the findings through academic means seemed appropriate. We therefore urge other researchers to look again at these and similar experiences with a view to obtaining further policy–relevant findings on factors leading to successful ORS scale–up.

As previously noted, ORS coverage rates in the 1980s were available for few countries, but have since improved with MICS and DHS [1]. Although we identified data sources for ORS coverage in 96 countries, these periodic surveys, one–time field studies, supply chain data and national surveys rely on caregiver recall of the illness episode and any treatment sought and provided. We selected a subset of all countries that have attempted to scale up ORS and for whom data were available at the time; it would be of interest to repeat the studies, drawing from a wider selection of country experiences, and to validate the findings of our quantitative analysis by ensuring that more than one assessor scores the various country factors.

Finally, we relied on impressions from key informants, who undoubtedly had extensive knowledge of the historical contexts, the actors involved and the issues. We cannot rule out the potential reporting bias inherent in the role that the key informants or their organizations played in promoting ORS use. We did not maintain tape recorded interviews, as we suspected that key informants would not be as open to sharing their perceptions if they were being recorded. Although we developed one interview guide, given the semi–structured nature of the interviews interview questions were not completely standardized across countries.

## CONCLUSIONS

This study was an in–depth effort to objectively gather and compare the limited data available on the factors associated with successful and unsuccessful scale–up of ORS programs. We identified a number of characteristics of successful ORS scale–up programs, including involvement of a broad range of key players, addressing supply and demand generation together, and working with both public and private sectors. Failure to involve key partners will often result in major gaps in the scale–up plan and critically affect sustainability. We found that the cost of scale–up efforts has not historically been well–documented. There are implications for the way that future ORS scale–up efforts should be directed to avoid some of the mistakes of the past. Efforts must synchronize demand generation, private provider outreach and public sector work. Rigorous monitoring and evaluation plans to track program impacts should be developed to address the gap in knowledge within the global community and improve our understanding of cost and sustainability. Future studies revealing lessons from other country experiences could also contribute to efforts to scale–up access to ORS and ultimately improve the lives of children who benefit the most from these efforts.

## 

**Table:** ORS – oral rehydration solution, U5 – under 5 years of age, DTP3 – Diphtheria–tetanus–pertussis, USAID – United States Agency for International Development, IMCI – Integrated Management of Childhood Illness, No.- number, Y/N – yes/no
