# Supplementary material for: Scaling up access to oral rehydration solution for diarrhea: Learning from historical experience in low– and high–performing countries
Source: J Glob Health. 2013 Jun;3(1):010404. doi: 10.7189/jogh.03.010404 (PMC3700030; doi:10.7189/jogh.03.010404)
Supplement: Online Supplementary Document [file jogh-03-010404-s001.pdf]

Online Supplementary Document
Wilson et al. Scaling up access to oral rehydration solution for diarrhea: Learning from historical experience in low- and high-performing countries.
Journal of Global Health 2013;3:010404

BACKGROUND

We are trying to understand why ORS/Zinc use to treat childhood diarrhea has been successfully scaled-up in some countries, but not in others. We are developing a series of country-specific case studies in order to compare successful scale up programs, such as in Bangladesh where ORS is widely available and used to treat most cases of childhood diarrhea, with countries where ORS is not widely available and/or successful scale-up was not sustained.

We have read publicly available documents, but there are still substantial gaps in our knowledge of the local characteristics of health delivery, the history of ORS and zinc scale up programs, and big picture insights into the conditions that favor ORS/Zinc scale up. We are hoping that you might be able to help us fill in some of those gaps or might suggest additional people to talk to.

Also, to the extent that we still have questions, we hope that you might be able to suggest additional people that we can contact who might be able to provide the information we're looking for.

Probing questions –

Do they understand what we are trying to do and do they have any immediate feedback?

PERSONAL EXPERIENCE QUESTIONS

Please tell us about your background with regards to child health and <country>.

What is your experience with the ORS/Zinc scale up program in <country>?

GENERAL QUESTIONS

1. What is your brief (two-minute) history of ORS in <country>?

2. What were the major drivers of the success/lack of success of ORS scale-up?

3. Who was involved in the delivery of diarrhea control programs?

4. What would it take, in your opinion, to sustain ORS coverage at greater than 60% and to get zinc to reach 25% coverage within the next 10 years in <country>?

5. What are the top factors you believe led to a (un)successful scale-up? Why?

6. Looking back, is there something that you would have changed (or done differently)?

MANUFACTURING AND SUPPLY

1. How do people in <country> get ORS (and zinc)?

2. Do you think there is a gap between knowledge of ORS and use of ORS?

    a. If so, is that lack of education? Marketing? Other? What do you think needs to be done to decrease that gap?

    b. If not, what was done to bridge the gap between knowledge and use?

3. Do you know how much of the population is using homemade ORS, rather than the pre-packaged version?

    a. If any, do you think this is alright? Should there be steps taken to reduce that even further?

    b. Do you think the price of the ORS sachets is prohibitive to any population groups? How much cheaper is making a home-made solution than buying the packet? Does this influence some people to make at-home solutions?

    c. How would you describe homemade ORS? Ingredients? Perceptions?

4. Are there any segments of the population that do not have access to ORS?

    a. Which ones? Why? What do you think needs to be done to increase access?

MARKETING AND EDUCATION

1. How did/do people (child caregivers, public providers, private providers) learn about ORS (and zinc)?

2. In your opinion, what has kept demand for ORS at its current level (high/low)? Marketing to consumers, education of healthcare providers, cultural acceptance, other? <lack of these factors for countries with low ORS demand>

GOVERNMENT AND DONOR SUPPORT

1. Where did the funding for the scale-up program come from?

2. What were the biggest expenses of the project?

3. What social or political aspects of <country> do you think have made it possible to scale-up and promote ORS and zinc?

ADDITIONAL SOURCES

1. Do you have any suggestions for anyone else that would be useful to speak to with respect to the ORS zinc scale-up activities?
